# Oral cancer via the bargain bin: The risk of oral cancer associated with a smokeless tobacco product (*Naswar*)

**DOI:** 10.1371/journal.pone.0180445

**Published:** 2017-07-10

**Authors:** Zohaib Khan, Steffen Dreger, Syed Majid Hussain Shah, Hermann Pohlabeln, Sheraz Khan, Zakir Ullah, Basheer Rehman, Hajo Zeeb

**Affiliations:** 1 Leibniz Institute for Prevention Research and Epidemiology - BIPS, Bremen, Germany; 2 Health Sciences Bremen, University of Bremen, Bremen, Germany; 3 Ayub Medical College-Dental Section, Abbottabad, Pakistan; 4 Khyber Medical University, Peshawar, Pakistan; 5 Khyber Teaching Hospital, Peshawar, Pakistan; 6 Khyber College of Dentistry, Peshawar, Pakistan; Università degli Studi della Campania "Luigi Vanvitelli", ITALY

## Abstract

In the wake of smokeless tobacco (SLT) being advocated as a mean of tobacco harm reduction, it is pertinent to establish individual health risks associated with each SLT product. This case-control study was aimed at assessing the risk of oral cancer associated with a smokeless tobacco product (*Naswar*). The study was conducted from September 2014 till May 2015 in Khyber Pakhtunkhwa, Pakistan. Exposure and covariate information was collected through a structured questionnaire. Conditional logistic regression was used to calculate odds ratios (OR) along with their 95% confidence intervals (CI). 84 oral cancer cases (62% males) and 174 age- and sex-matched controls were recruited. Ever users of *Naswar* had more than a 20-fold higher risk of oral cancer compared to never-users (OR 21.2, 95% CI 8.4–53.8). Females had a higher risk of oral cancer with the use of *Naswar* (OR 29.0, 95% CI 5.4–153.9) as compared to males (OR 21.0, 95% CI 6.1–72.1). Based on this result, 68% (men) and 38% (women) of the oral cancer burden in Pakistan is attributable to *Naswar*. The risk estimates observed in this study are comparable to risk estimates reported by previous studies on other forms of SLT use and the risk of oral cancer in Pakistan. The exposure-response relationship also supports a strong role of *Naswar* in the etiology of oral cancer in Pakistan. Although still requiring further validation through independent studies, these findings may be used for smokeless tobacco control in countries where *Naswar* use is common.

## Introduction

Oral cancer is one of the most common cancers in the world with approximately 300,000 incident cases each year [1]. Pakistan has one of the highest prevalence of oral cancer in the world [2]. With an age-standardized incidence rate of 9.8/100,000, oral cancer has become the most frequent cancer among males and the second most common cancer among both sexes in Pakistan [1]. A variety of risk factors like diet, alcohol, tobacco use, infections, genetic and environmental factors are associated with oral cancer. Among these, tobacco smoking and alcohol use have been widely researched and are universally considered as causal factors [3]. Smokeless tobacco (SLT) is labeled as carcinogenic by the World Health Organization [4]. Studies from South Asia have established SLT as a risk factor for oral cancer [5,6], but some investigations from industrialized countries, particularly Sweden, where SLT use is common, do not show an increased risk of oral cancer linked to the use of some SLT products [7,8]. These conflicting results become particularly important in the light of SLT products being considered as an alternative to smoking [8], and as means of harm reduction [9–11].

An estimated 250 million people use smokeless tobacco in South Asia [12]. Research on SLT products and the risk of oral cancer in South Asia has traditionally focused on Betel-quid and Gutkha [5,6]. This is understandable, as the majority of SLT research has been carried out in India, where the most common forms of SLT are Gutkha and Betel-quid [13]. *Naswar* is a mixture of dried tobacco leaves, ash, lime and flavoring agents [14]. It is kept in the buccal sulcus of the mouth and the active agents are absorbed through the oral mucosa. *Naswar* use is often associated with the Pashtun tribes of Afghanistan and Pakistan but is also used in Central Asia, India, Bangladesh and by expat communities of these countries across the world [15].

*Naswar* is a much cheaper product compared to cigarettes. An average pack of *Naswar* costs approximately a 10th of the price of a cigarette pack in Pakistan and as such is gaining popularity as a cheap alternative to smoking [16]. It is also being advocated as a cheaper nicotine replacement therapy for people trying to quit smoking [17]. The sale and manufacture of *Naswar* in Pakistan are not regulated [18], and the sizing of the package and the constituents vary from one manufacturer to the other. Thus, the amount of carcinogenic agents also differs among the different brands available on the market [14]. Unlike cigarettes, the individual serving size also varies and is dependent upon personal preferences. This renders the correct establishment of the magnitude of the risk of oral cancer associated with a discernable *Naswar* “dose”, particularly challenging. A few studies from the south of Pakistan, where other forms of SLT are more popular [19,20], have reported risk estimates for oral cancer associated with *Naswar*, but there is scanty evidence from the Khyber Pakhtunkhwa province (KPK), which has the highest number of *Naswar* users in Pakistan [21]. The dearth of evidence needed to establish *Naswar* as carcinogenic for humans has also been acknowledged by the International Agency for Research on Cancer in its monograph on smokeless tobacco [22].

Given the conflicting research findings on the risks of SLT use and the scarcity of research on assessment of *Naswar* as being carcinogenic to humans, we carried out a case-control study in the KPK to assess the association between *Naswar* and the risk of oral cancer. We particularly focused on exposure quantification by using a novel method of *Naswar* pack-years (NPY), assessment of exposure-response relationships and gender stratified risks. We also assessed the fraction of incident oral cancer among the study population that can be attributed to *Naswar*.

## Methods

### Study design and setting

A multi-center matched case-control study was carried out in two major cities of the Khyber Pakhtunkhwa province of Pakistan between September 2014 and May 2015. Peshawar is the capital city of the province; while Abbottabad is considered as the summer capital. The province has an area of 74,521 sq km and a total population of 17.5 million. The population of Peshawar is 3,575,000, while that of Abbottabad is 1,182,000. The majority of the population lives in rural areas and agriculture and trade are the main earning resources. Cases were recruited at three tertiary care centers (Maxillofacial Surgery department of Khyber College of Dentistry, Peshawar, Ear, Nose, and Throat department of the Khyber Teaching hospital, Peshawar and the Maxillofacial Surgery Department of Rehmat Memorial Hospital, Abbottabad). Since primary and secondary healthcare facilities in the province do not have adequate means to diagnose and/or manage oral cancer patients, the included study centers are mainly responsible for the provision of both diagnostic and curative services for oral cancer. The catchment area of the study centers includes the whole province along with the Federally Administered Tribal Areas (FATA) of Pakistan. Controls were recruited from the same centers as well as from two additional health facilities in Peshawar (Pakistan Paraplegic Center, Peshawar and Institute for Physical Medicine and Rehabilitation, Khyber Medical University, Peshawar). These facilities also provide health services to the population of the whole province. All study centers were selected based on expert opinions from local cancer physicians and dentists. The recruitment was carried out for a nine-month period starting September 2014 and ending in the first week of June 2015.

### Power calculation

The study size for a case-control ratio of a 1:1 and 1:2, was calculated in Epi Info 7 by using the Fliess method with continuity correction factor. The prevalence of *Naswar* (15%) among the general population (controls) was derived from a nationally representative survey[21]. To detect an OR of 3.0 with a two-sided 95% confidence level and a power of 90%, we had to recruit 78 cases and 156 controls.

### Ethical approval

Ethical approval for the study was granted by the ethical review board of Khyber Medical University and also by the ethical review committee of Khyber College of Dentistry. Written approvals to carry out the study were also obtained from the heads of the participating centers. Written consent was taken from each study participant before the interview and subsequent collection of biosamples. All study participants had the option to retract their consent at any stage of the study if they did not want to be a part of the study. To ensure maximum participation, laboratory charges related to the histopathological diagnosis and confirmation of the presence of oral cancer were borne from the study fund. These charges are normally paid out of pocket by the patients.

### Recruitment of cases and controls

#### Cases

Potential cases were recruited based on a clinical differential diagnosis of oral cancer. For the purpose of this study, “oral cancer” was defined as squamous cell carcinoma of the buccal mucosa, lip, tongue and the oropharynx: The ICD-10 classification was used to designate oral cancer sites to be included in the study. The eligible sites included lip, the base of tongue, other and unspecified parts of the tongue, gum, floor of mouth, palate, other and unspecified parts of the mouth, tonsil, and oropharynx (C00—C06 and C09—C10). A potential case was confirmed as a “definitive case”, only after the histopathological confirmation of the presence of squamous cell carcinoma at one of the above-mentioned sites.

#### Controls

Subjects with any condition, except for cancer, pulmonary disease, cardiovascular disease, gastrointestinal disease and periodontal disease, were eligible to be recruited as controls because these diseases are known to be related to tobacco use. Two age (10-year bands) and sex-matched controls were recruited per case from the out-patient and in-patient departments of the study centers. Following are the inclusion and exclusion criteria for recruitment:

#### Inclusion criteria

- Only incident cases who had not yet undergone any treatment for oral cancer were included as cases;
- all included cases and controls were permanent residents and/or living in KPK or FATA for at least twelve months prior to the interview;
- a case or control was only included if he/she could provide an informed consent and was deemed physically fit to be interviewed by the resident doctor/s.

#### Exclusion criteria

- Subjects with tumors/malignancy of the hypopharynx, nasopharynx, and salivary glands, or who had previous treatment for oral cancer before the interview;
- subjects who were not permanent residents and/or had not been living in the Khyber Pakhtunkhwa province or the federally administered tribal areas for at least 12 months prior to the interview;
- unable to provide informed consent due to illness or deemed “physically not fit” for interview by a resident doctor.

#### Matching

Two controls per case, frequency-matched for age (10-year bands) and sex, were recruited for the study.

### Exposure variables

A Directed Acyclical Graph (DAG) analysis (S1 Fig, part a and b) was carried out to ascertain study variables for which data needed to be collected. Oral cancer was the main outcome and *Naswar* was the primary exposure variable. Age, sex, socioeconomic status (SES), tobacco smoking and alcohol use were determined as the Minimal Adjustment Set (MAS) i.e. confounding exposures. Additionally, data were collected for Betel-quid chewing, sunlight exposure, diet, oral hygiene habits and history of the systemic and oral disease.

### Data sources/measurement

Data on the study variables was collected through a structured questionnaire adapted from a large European case-control study on upper aero-digestive tract cancers [23]. Face to face, interviews were conducted with both cases and controls. Apart from questions about the “current illness”, the questionnaire used for both groups was the same.

#### Naswar use

Data on ever use, daily frequency, total duration in years, duration of single use and type of *Naswar* were recorded. To determine the cumulative exposure to *Naswar*, we developed a novel measure of “*Naswar* pack-year (NPY)”.

#### Tobacco smoking

Data regarding ever smoking, past smoking, current smoking, frequency and total duration of use in years for cigarettes and/or water pipe were recorded.

#### Alcohol drinking

Although alcohol is an established risk factor for oral cancer and can modify or confound the effects of other risk factors, the section of the questionnaire on alcohol use was considerably shortened from the one in the ARCAGE study and had only six questions. This was because alcohol use is forbidden in Islam, the main religion in this region, and is also a culturally and socially unacceptable habit in Pakistan. This renders any talk about alcohol as a taboo. However, we still collected data on ever and never use of alcohol and total duration of alcohol use in order to account for the effects of alcohol use, if any, during analysis.

#### Socioeconomic status

SES was assessed using a simple poverty scorecard developed for Pakistan[24]. The scorecard is used to determine the probability of a household to be situated above the national poverty line i.e. a higher score means a higher probability of being placed above the national poverty line and vice versa. This method has been previously used in social science research in Pakistan but never in health research. The scorecard consists of ten close-ended questions pertaining to assets, education, job type, the number of children, and source of drinking water. The responses are marked and scored according to pre-determined scores. The overall score is then translated into the likelihood of a household being below or above the national poverty line. The advantage of this approach is that it is based on household-level data, which is cognizant of the Pakistani culture of joint families.

Dietary habits were assessed using a food frequency questionnaire, containing questions about meat, vegetables, fruit and tea intake. The intake was recorded in terms of frequency per month. The oral health section included questions regarding frequency and mode of mouth and/or teeth cleaning along with the presence of oral disease and the use of dentures. History of disease, such as candidiasis, herpes, warts and regurgitation, was recorded in the systemic disease section. Pictures were used to aid the memory of participants. Sun exposure was assessed by asking questions about the average time spent in the sun during a day. Questions regarding any means of sun protection used by the participants were also included.

### Exposure quantification

#### Age

Age was categorized into ten-year bands.

#### SES

Based on the probability of lying above the national poverty line, we assigned our study participants into three categories: high (probability > 66%), medium (34% - 66% probability) and low (probability < 34%).

#### Habits

An “Ever user” of *Naswar*, cigarette, betel-quid, water pipe, or alcohol was defined as a person who had practiced the habit at least once per week for one year in his life, consequently a person who had never used the above or had only used them with a frequency of less than once per week for an year was defined as a “Non-user”. A “current user” of *Naswar* was defined as someone who has been using *Naswar* at least once per week in the 12 months preceding the interview, including those who had stopped the habit within those 12 months. A “past user” was defined as a person who had used *Naswar* at least once a week for a year but had quit the habit before the 12 months preceding the interview.

### Naswar-pack-years

Naswar production is not regulated in Pakistan and therefore the correct assessment of exposure categories and dose-responses is very difficult. Usually, the packages come in different sizes and the size of individual serving depends on users and varies to a great extent based on personal preference. To address this issue, a selected sample of 50 case and control participants, who were *Naswar* users, were asked to make a serving of *Naswar*, similar in size, to what these participants had been or were currently using. These servings were weighed and the average weight of a single serving was calculated. We also acquired 62 different *Naswar* packages from the 23 districts of the KPK, the capital city of Pakistan and the five provincial capitals, and calculated the average weight of these *Naswar* packages. The number of servings/package was computed by dividing the average weight of a package by the average weight of a serving. From these data, NPY were calculated by using the formula

(Number of servings per day ×Total duration of Naswar habit in years) /(Number of servings per Naswar packet)

The average weight of a Naswar pack was 43.6 g (95% CI: 42.2–45.7 g). The average weight of a Naswar pellet was 2.1 g (95% CI: 2.0–2.3 g). The number of pellets per Naswar package was 20.6. Conservatively, a Naswar pack-year was thus defined as 20 pellets of Naswar used per day for one year. For the conditional model, NPY was categorized into 4 categories i.e. None, 1–10, 11–20 and more than 20, the intensity of *Naswar* use (in minutes) was categorized into None, 1–5, 6–10 and greater than 10.

### Bias reduction

The study participants were blind to the main research hypothesis. Interviewers and cases were partially blind to the case status of the participants, as interviews with the cases took place before a definitive diagnosis had been established. This approach helped us reduce temporal ambiguity and differential recall bias among cases and controls. Recruitment of incident-only cases was aimed at avoiding recall bias, as well as the prevalance-incidence bias, where selective survival may have resulted in an under or over representation of exposure in the prevalent cases.

### Statistical methods

Data were entered and stored in Epi Info 7 [25]. The analysis was carried out in SAS version 9.3 [26]. Crude odds ratios (OR^1^) along with their 95% CI were calculated using conditional logistic regression (conditioned for age and sex). Moreover, adjusted odds ratios were derived (OR^2^), taking simultaneously into account the MAS of variables. We also calculated the population attributable fraction (PAF) for KPK and Pakistan, using the OR from the conditional logistic regression model and prevalence of *Naswar* (p) use from a nationally representative tobacco prevalence surveys from Pakistan [21, 27] by using the formula: $PAF=\frac{\left[\text{p}\left(\text{OR-1}\right)\right]}{\left[\text{p}\left(\text{OR-1}\right)\text{+1}\right]}$. The total number of attributable incident cases (AC) of oral cancer was obtained by the formula AC = PAF * TC, TC is the total number of annual incident cases of oral cancer. The estimated annual number of incident cases of oral cancer in Pakistan was extracted from Globocan, 2012 [1].

## Results

### Participants profile

Based on our initial sample size calculation, we had to recruit 107 cases and 107 controls for a 1:1 case/control ratio, or 78 cases and 156 controls for a 1:2 case/control ratio. The study initially started with a ratio of 1:1 among cases and controls. However, in December 2014, Peshawar saw a deadly terrorist attack killing almost 150 children and resulting in a very tight security situation in the whole province. The uncertain security situation led to a decrease in patient in-flow at most hospitals in Peshawar city as both inter and intra-city movement came to a halt. The security situation and the resulting decrease in patient in-flow hampered recruitment of cases in Peshawar making it difficult to reach the desired number of 106 cases for the study. Therefore, in February 2015, it was decided to recruit two controls per case in order to be able to achieve the desired power for the study.

A total of 88 potential cases and 179 age and sex-matched controls were asked to participate in the study. 86 cases and 174 controls agreed to participate, The participation rate was 98% for cases and 96% for controls. The final sample included 84 cases and 174 age and sex-matched controls (Table 1) as two cases were excluded from the analysis because they had a cancer type other than squamous cell carcinoma. The majority of cases were males (n = 52). The mean age of male cases and controls was 56.3 (±13.0) and 57.4 (±12.7) years, respectively. Among females, the mean age of cases and controls was 51.4 (±14.4) and 57. 3 (±16.9) years, respectively. The male to female ratio was 1.7: 1 and about 34% (15 males, 14 females) of the cases were 50 years of age or younger 50.

**Table 1:** Distribution of cases and controls by study recruitment center.

	Study centers					
Study status	KCD	KTH	RMH	PPC	KMU	Total
**Cases *n* (%)**	57 (67.8)	9 (10.7)	18 (21.4)			84
**Controls *n* (%)**	63 (36.2)	36 (20.6)	20 (11.4)	28 (16)	26 (14.9)	174

^KCD: Khyber College of Dentistry, KTH: Khyber Teaching Hospital, RMH: Rehmat Memorial Hospital, PPC. Pakistan Paraplegic Center, KMU: Khyber Medical University^

The most common primary sites of oral carcinoma tumors were the gums (n = 37) and the buccal mucosa (n = 24). Histologically, 75% of the tumors were “well-differentiated”, 17% were “moderately differentiated”, and the remaining tumors either poorly differentiated or “undifferentiated”. From a total of 23 districts in the Khyber Pakhtunkhwa province, only two were not represented among the cases. Peshawar being the most populous city of the province had the highest number of cases. Six cases originated from the federally administered tribal areas. The distribution of MAS variables among the participants overall and stratified by sex is provided in Table 2. *Naswar* was the most prevalent habit among both cases (79.7%) and controls (27.5%). The majority of the participants (95% cases, 92% controls) belonged to the low or medium SES strata. Initial univariate analysis (chi-square tests) revealed that *Naswar* use, smoking, and sex were significantly (p<0.05) associated with oral cancer.

**Table 2:** Distribution of the lifestyle risk factors for oral cancer, by sex, among cases (n = 84) and controls (n = 174) in Khyber Pakhtunkhwa, Pakistan.

Risk Factors	Males		Females		Total	
	Cases	Controls	Cases	Controls	Cases	Controls
	n (%)	n (%)	n (%)	n (%)	n (%)	n (%)
**Naswar**						
Never	3 (5.7)	64 (59.2)	14 (43.7)	62 (93.9)	17(20.2)	126 (72.4)
Ever	49 (94.2)	44 (40.7)	18 (56.2)	4 (6.1)	67 (79.7)	48 (27.5)
Current	34 (65.3)	28 (25.9)	12 (37.5)	0 (0.0)	46 (54.6)	28 (16.1)
Past	15 (28.8)	16 (14.8)	6 (18.7)	4 (6.1)	21 (25.1)	20 (11.4)
**Cigarette Smoking**						
Never	29 (55.7)	82 (75.9)	27 (84.3)	65 (98.4)	56 (66.6)	147 (84.4)
Ever	23 (44.3)	26 (24.1)	5 (15.6)	1 (1.6)	28 (33.3)	27 (15.5)
**Betel-quid Chewing**						
Never	50 (96.1)	108 (100.0)	30 (93.7)	66(100)	80 (95.2)	174 (100)
Ever	2 (3.8)	0 (0.0)	3 (6.2)	0 (0.0)	4 (4.7	0 (0.0)
**Water-pipe smoking**						
Never	48 (90.3)	107 (99.1)	30 (93.7)	66 (100)	77 (91.6)	173 (99.4)
Ever	4 (9.69)	1 (0.9)	3 (6.2)	0 (0.0)	7 (8.3)	1 (0.6)
**Alcohol**						
Never	49 (94.2)	105 (97.2)	31(96.8)	66 (100.0)	80 (95.2)	171 (98.2)
Ever	3 (5.7)	3 (2.7)	1 (3.1)	0 (0.0)	4 (4.7)	3 (1.7)
**Socio-economic status**						
Low	26 (50.0)	50 (47.2)	13 (40.6)	16 (22.7)	39 (46.4)	66 (37.9)
Medium	24 (46.1)	51 (48.1)	17 (53.1)	43 (63.6)	41 (48.8)	94 (54.0)
High	2 (3.8)	7 (5.6)	2 (6.2)	7 (13.6)	4 (4.7)	14 (8.0)

### Main results

Table 3 shows the univariate as well as the simultaneously adjusted risk estimates for different risk factors among the study participants. Ever and current *Naswar* users had a more than 20-fold risk increase of oral cancer compared to non-users (ever: OR 21.2, 95% CI 8.4–53.8), (current: OR 27.4, 95% CI 10.0–74.7). Ever smoking also doubled the risk of oral cancer, compared to non-smokers (OR 2.2, 95% CI 1.4–4.5), while alcohol consumption was not significantly related to the risk of oral cancer (p-value = 0.19). In general, a higher SES was associated with a lower risk for oral cancer; however, this finding was also not significant (p-value = 0.36). Tables 4 and 5 provide an overview of the risk of oral cancer associated with *Naswar* stratified by males and females, respectively.

**Table 3:** Risk of oral cancer associated with the lifestyle risk factors among both sexes (84 cases, 174 controls) in Khyber Pakhtunkhwa, Pakistan, derived from conditional logistic regression (conditioned on age and sex).

Risk Factors	Casesn (%)	Controls n (%)	OR^1^ (95% CI)	OR^2^ (95% CI)
**Socio-Economic Status**				
Low	39 (46.4)	66 (37.9)	1.0	1.0
Medium	41 (48.8)	94 (54.1)	0.7 (0.4–1.2)	0.7 (0.4–1.3)
High	4 (4.8)	14 (8.0)	0.5 (0.1–1.5)	0.5 (0.1–1.7)
**Smoking**				
Never	56 (66.6)	147 (84.4)	1.0	1.0
Ever	28 (33.3)	27 (15.5)	3.0 (1.5–5.8)	2.2 (1.4–4.9)
**Alcohol**				
Never	80 (95.2)	171 (98.2)	1.0	1.0
Ever	4 (4.7)	3 (1.8)	2.7 (0.6–12.1)	0.7 (0.1–4.1)
**Naswar**				
Never	17 (20.2)	126 (72.4)	1.0	1.0
Ever	67 (79.7)	48 (27.5)	22.9 (9.2–57.4)	21.2 (8.4–53.8)
Current	46 (54.7)	28 (16.1)	28.0 (10.5–74.0)	27.4 (10.0–74.7)
Past	21 (25.0)	20 (11.4)	16.4 (5.8–46.7)	14.3 (4.9–41.2)
**Naswar Pack Years**				
0	17 (20.2)	126 (72.4)	1.0	1.0
1–10	16 (19.0)	16 (9.1)	15.3 (5.2–44.9)	12.5 (4.1–38.0)
11–20	27 (32.1)	15 (8.6)	28.7 (9.9–82.8)	26.5 (9.0–78.2)
>20	24 (28.5)	17 (9.7)	28.3 (9.3–86.2)	28.9 (9.3–90.2)
**Naswar dip duration (minutes)**				
0	17 (20.2)	126 (72.4)	1.0	1.0
1–5	19 (22.6)	39 (22.2)	8.5 (3.1–23.3)	7.2 (2.5–20.4)
6–10	23 (27.3)	6 (3.4)	67.6 (18.6–245.6)	61.8 (16.6–229.5)
>10	25 (29.7)	3 (1.8)	142.2 (31.1–650.5)	136.2 (29.1–638.2)
**Naswar Saliva** *				
Swallow	20 (29.8)	8 (20.8)	1.0	1.0
Spit	47 (70.1)	40 (79.1)	0.4 (0.1–1.3)	0.4 (0.1–1.4)
**Naswar Type**				
Non-user	17 (20.2)	126 (72.4)	1.0	1.0
Black	50 (59.5)	37 (21.2)	22.2 (8.6–56.7)	21.3 (8.2–55.4)
Green	17 (20.2)	11 (6.3)	25.9 (8.0–83.0)	21.0 (6.4–68.9)

*_ Ever users only;_

_OR_^1^_: Basic model conditioned for age and sex;_

_OR_^2^_: Basic model adjusted for other MAS variables._

**Table 4:** Naswar use and the risk of oral cancer among men (52 cases,108 controls) in Khyber Pakhtunkhwa, Pakistan, crude and adjusted risk estimates from simple logistic regression.

Risk Factor	Cases		Controls		OR^1^ (95% CI)	OR^2^ (95% CI)
	n	%	n	%		
**Naswar habit**						
Never	3	5.8	64	59.3	1.0	1.0
Ever	49	94.2	44	40.7	23.7 (6.9–81.0)	21.0 (6.1–72.1)
Current	34	65.4	28	25.9	25.9 (7.3–91.4)	23.4 (6.6–82.1)
Past	15	28.8	16	14.8	20.0 (5.1–77.5)	16.4 (4.1–65.4)
**Naswar Pack Years**						
0–10*	12	23.1	78	72.2	1.0	1.0
11–20	20	38.5	13	12.0	9.9 (3.9–25.2)	9.6 (3.6–25.5)
>20	20	38.5	17	15.7	7.6 (3.1–18.5)	8.7 (3.3–22.6)
**Dip duration (minutes)**						
0–5**	14	26.9	100	92.6	1.0	1.0
6–10	20	38.5	5	4.6	28.5 (9.2–88.3)	23.0 (7.4–71.5)
>10	18	34.6	3	2.8	42.8 (11.1–164.3)	39.7 (9.9–158.5)
**Naswar type**						
Green	11	21.2***	10	9.3	1.0	1.0
Black	38	73.1***	34	31.5	1.0 (0.3–2.6)	1.0 (0.3–2.8)

*, **Includes “Never users”,

*** Ever users only,

OR1: Crude Odds Ratio,

OR2: Adjusted for age, SES, smoking, and alcohol, CI: Confidence _Interval._

**Table 5:** Crude and adjusted risk estimates for oral cancer associated with Naswar use among women (32 cases, 66 controls) in Khyber Pakhtunkhwa, Pakistan, derived from simple logistic regression.

Variables	Cases		Controls		OR^1^ (95% CI)	OR^2^ (95% CI)
	N	%	n	%		
**Naswar habit**						
Never	14	44.1	62	93.9	1.0	1.0
Ever	18	55.9	4	6.1	19.9 (5.8–68.1)	29.0 (5.4–153.9)
**Naswar Pack Years**						
0–10*	21	65.6	64	97.0	1.0	1.0
>10	11	34.4	2	3.0	16.7 (3.4–81.8)	16.0 (2.7–93.7)
**Dip duration (minutes)**						
0	14	43.5	62	93.5	1.0	1.0
1–5	8	25.0	3	4.5	11.8 (2.7–50.2)	16.7 (2.2–124.1)
>5	10	31.3	1	1.6	44.2 (5.2–374.8)	50.2 (5.1–495.9)
**Naswar type**						
Green	6**	18.8	1	1.5	1.0	1.0
Black	12	37.5	3	4.5	0.6 (0.1–11.5)	0.8 (0.1–11.5)

*_ Includes “Never users”,_

**_ Ever users only,_

_OR1: Crude Odds Ratio, OR2: Adjusted for age, SES, smoking and alcohol, CI: Confidence Interval_

The overall PAF of *Naswar* for oral cancer in Pakistan was 59%. The sex-specific PAF of *Naswar* for oral cancer in Pakistan was 68% and 38% for males and females, respectively. The PAF was 75% for KPK. Sex-specific PAF for KPK was not calculated due to lack of data. The total number incident cases of oral cancer in both sexes in Pakistan attributable to *Naswar* (AC) was 9,094 (15,414 total incident oral cancer cases in Pakistan.).

## Discussion

### Statement of main findings

*Naswar* contributes to about 70% of oral cancers in the study region. Ever users of *Naswar* were more than 20 times likely to develop oral cancer compared to non-users. Compared to non-users and participants with a comparatively low cumulative exposure (NPY) to *Naswar* (<11), both male and female ever-users with a higher NPY count had a significantly higher risk of oral cancer. A similar relationship was seen with the intensity of exposure between both sexes, with a significant increase in risk among *Naswar* users who kept *Naswar* in the mouth for more than five minutes as compared to non-users and participants who kept *Naswar* in their mouth for a shorter duration.

### Interpretation and generalizability

Smokeless tobacco is considered as a risk factor for oral cancer and its use is on a rise globally and in particular in South Asian countries [28,29]. *Naswar* has not been researched extensively, particularly in the context of cancer risk. A previous study from the KPK reported a high biochemical risk of cancer associated with the constituents of *Naswar* [14], the present study conducted in the same region provides epidemiological evidence to further strengthen that argument. A more than 80% prevalence of *Naswar* use among the oral cancer cases in our study is comparable to previous findings from the same region [30,31]. The prevalence of *Naswar* use among the controls at 27% is comparable to previous findings (31%) about *Naswar* use in Peshawar [32], yet substantially higher than the national figure of 7.3% [21]. The difference can be explained by the stark disparity in tobacco consumption practices among the different provinces of Pakistan. While the national figures are based on a representative sample of all the provinces of the country, our sample consists of subjects belonging to KPK only, where *Naswar* use is almost like a cultural practice [33].

We report a very high magnitude of risk for oral cancer associated with the use of *Naswar*. This finding is consistent with that of other studies from India and Pakistan on the risk of oral cancer associated with the use of other forms of SLT such as Gutkha and Betel-quid [5,6]. However, in our study, the observed risk estimates are even higher compared to those associated SLT products. A plausible explanation for this risk difference might be a comparatively higher amount of “Tobacco-Specific Nitrosamines” and nicotine, and a lime induced higher alkalinity (pH) of *Naswar* compared to Gutkha and Betel-quid [34]. Nicotine causes dependence and a higher nicotine level coupled with a high pH can cause stronger cravings and more frequent and/or prolonged use of the SLT products [35], leading to a stronger exposure to the carcinogenic agents. There are also suggestions that *Naswar* causes local tissue trauma by erosion [36], and chronic tissue trauma is an independent risk factor for cancer [37]. The ash and lime used in the preparation of *Naswar* may also be contributing a high level of toxins and heavy metals to the composition, thus adding to its potential toxicity [14].

Some previous studies from Southern Pakistan have reported risk estimates for oral cancer and *Naswar* which are lower in magnitude than the risk estimates we report [19,20]. This difference may be attributed to the diverse SLT consumption practices in different parts of Pakistan. Betel—quid use is very common in the south, while *Naswar* is mostly used in the north of Pakistan, including our study region [33]. In our study, Betel-quid use was not significantly related to an elevated risk of oral cancer and the prevalence of Betel-quid use was much lower than previous reports [19,20]. Furthermore, a large case-control study from Pakistan carried out in the 1970s [19], reported a relative risk of 20 for oral cancer with the use of Nass (= *Naswar*), consistent with our findings. However, this study had some methodological limitations [38].

Our results show that current users of *Naswar* had a higher risk compared to past users. This finding is in line with those of a cohort study on SLT use and the risk of oral carcinoma from India [39]. The results of our exposure-response analysis are in accordance with those reported in independent studies as well as systematic reviews of literature from South Asia, where an increasing frequency, duration, and intensity of exposure were all related to a subsequent increase in the risk of oral cancer [5,6]. We have reported a higher adjusted OR for the risk of oral cancer with the use of *Naswar* among females as compared to males. Other studies of SLT and its effects on oral cancer reported similar findings that may be explained by lower background risk of oral cancer among females and a greater potential for oral mucosal damage among women as compared to men [6,40]. Our study reports population attributable risks of *Naswar* for oral cancer comparable to those reported for other forms of SLT from other South Asian countries [6]. Notably, the PAF for KPK is considerably higher than the national PAF due to a higher prevalence of *Naswar* use in the province and signifies the importance of *Naswar* as a major risk factor for oral cancer in this population.

### Strengths and limitations of this study

This study may suffer from drawbacks inherent to retrospective study designs. The study sample, particularly the hospital controls, may not be representative of the general population of KPK. However, we adopted wide eligibility criteria for the inclusion of controls with regards to their diagnosed disease to avoid recruitment of subjects who might be very similar to each other in terms of exposure and belonging to a narrow subset of the whole population. For recruitment of the participants, we chose the largest tertiary care facilities and in the case of oral cancer patients, the only public sector centers where diagnosis and treatment of oral cancer are carried out. We obtained a high response rate among potential study subjects, which may be attributed to the payment of laboratory charges on behalf of the case subjects as an incentive, and cooperation from the hospital staff at the study centers, who motivated control subjects to participate. We managed to exceed the number of cases and controls estimated during the sample size calculation. However, we still had to collapse a few exposure-response categories during the sex-stratified analysis, due to a small number of participants. This shortcoming warrants larger epidemiological studies to strengthen the evidence provided by this study.

Although we frequency-matched each case to at least two controls, there have been recent suggestions in the literature that an unconditional logistic regression analysis may yield equal or more robust and efficient results for matched studies [41]. We did not find any large differences between the effect estimates yielded by the conditional and the unconditional analysis, both being highly elevated and suggestive of a causal link between *Naswar* and oral cancer. This is the first adequately powered case-control study to be carried out on the risk factors for oral cancer in the Khyber Pakhtunkhwa province and the use of a “simple poverty card”, utilization of causal diagrams and “Naswar pack-years” gives it novelty among other similarly designed studies on use of smokeless tobacco and the risk of oral cancer. Another important feature of the study was the partial blinding of the study cases, as they were only differentially diagnosed at the time of interview and hence not fully aware of their condition. This may have diminished selective recall bias among the cases.

### Policy and practice implications

These findings are highly relevant for South and Central Asia, where *Naswar* use is common. As prices of cigarettes soar, more people might take up products like naswar, because of their lower prices [42]. The lack of published evidence on health risks associated with SLT, such as *Naswar*, may also contribute to this. It is, therefore, pertinent to produce further local evidence to inform public policy, as findings from developed countries may not be applicable in the local context because of a difference in composition of SLT products, which may be responsible for the observed differences in risk of oral cancer and other diseases between industrialized and developing countries [43]. To the best of our knowledge, this study is one of a handful of case-control studies focusing on *Naswar* and the associated risk for oral cancer. Until larger cohort studies are carried out to further assess this risk, the evidence from this study may be used to inform SLT control policies in countries where *Naswar* is used. The use of *Naswar* pack-years could also be incorporated into research and clinical practice to assess future risks for oral cancer with the use of *Naswar*.

## Supporting information

S1 Fig (part a and b). Relationship between the study variables before and after adjustment for the minimal adjustment set. (PDF)

S1 Checklist STROBE checklist. (DOC)

## Abbreviations

CI: Confidence Interval
DAG: Directed Acyclical Graph
KPK: Khyber Pakhtunkhwa province
MAS: Minimum Adjustment Set
NPY: *Naswar* Pack-Year
OR: Odds Ratio
SLT: Smokeless Tobacco
