# Supplementary material for: Oral cancer via the bargain bin: The risk of oral cancer associated with a smokeless tobacco product (Naswar)
Source: PLoS One. 2017 Jul 10;12(7):e0180445. doi: 10.1371/journal.pone.0180445 (PMC5503251; doi:10.1371/journal.pone.0180445)
Supplement: S1 Fig — (PDF) [file pone.0180445.s001.pdf]

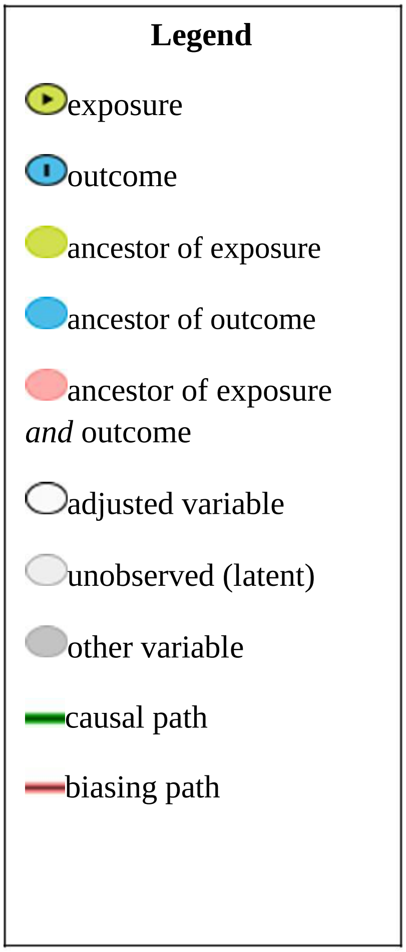

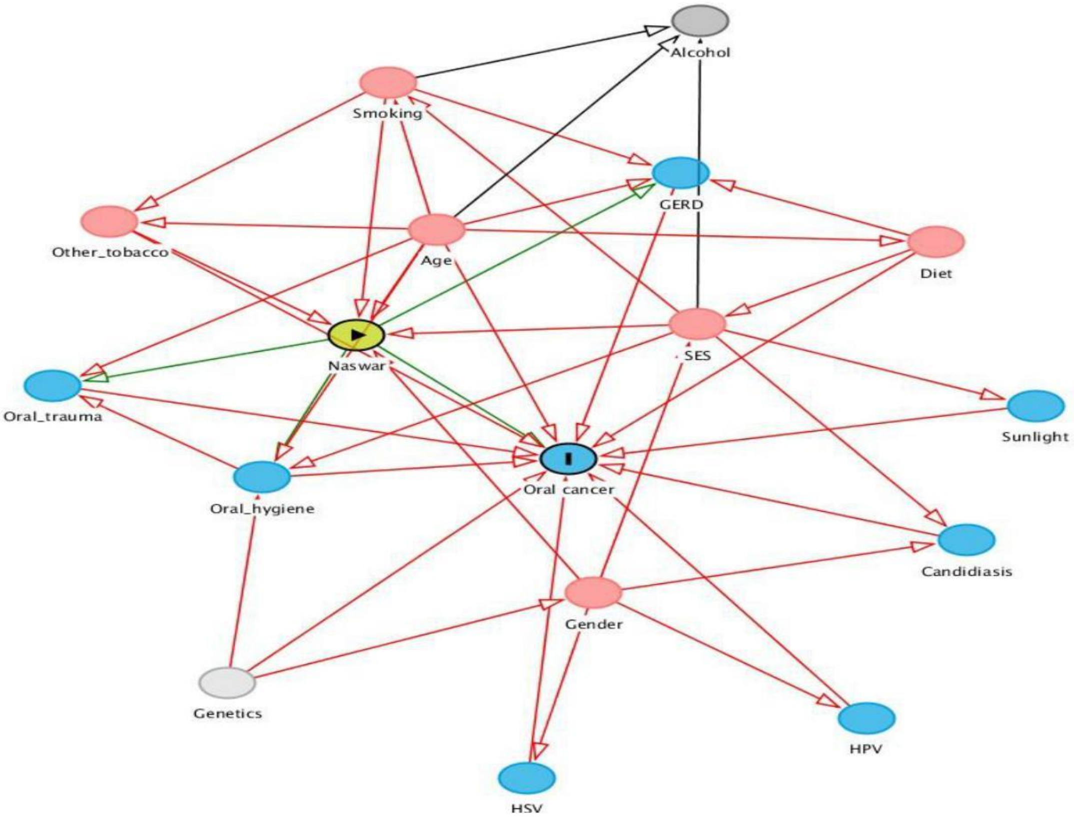

S1 Fig (a). Relationship between the study variables before adjustment for the minimal adjustment set.

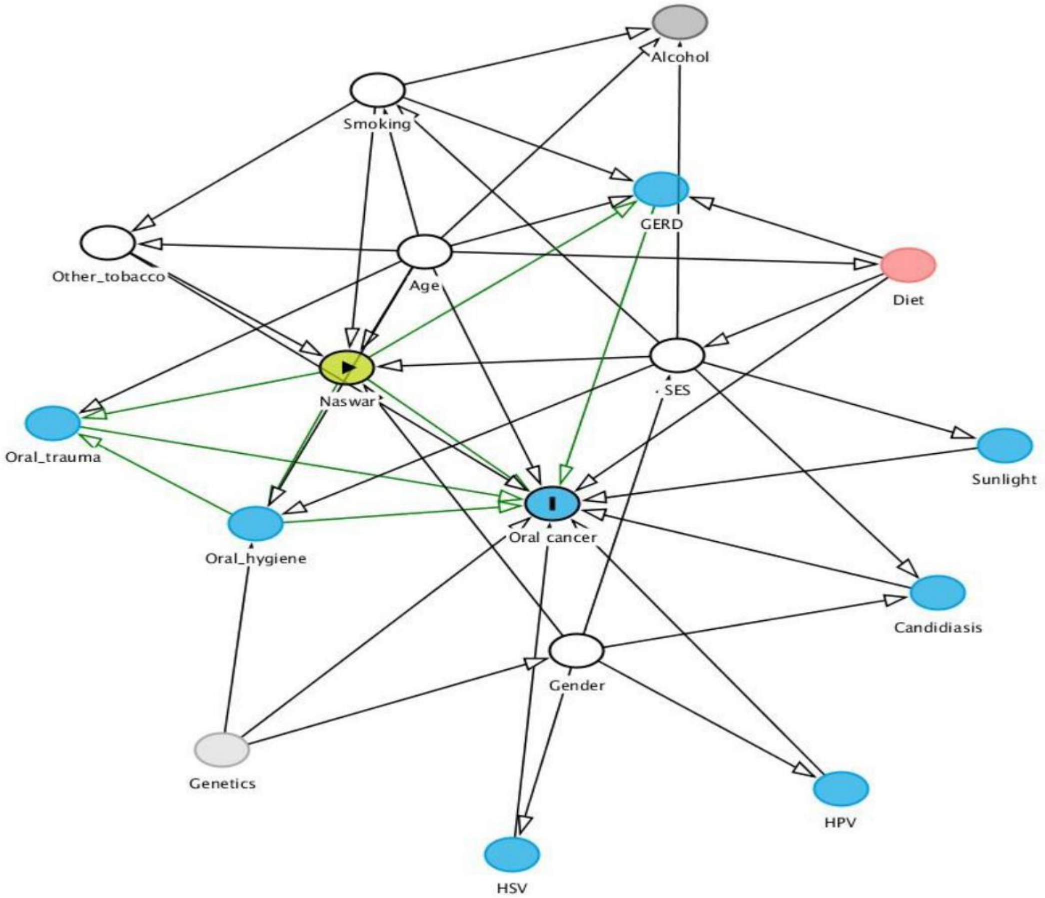

S1 Fig (b). Relationship between the study variables after adjustment for the minimal adjustment set.
